# Characterization of the complete mitochondrial genome of *Peniophora lycii* (Russulales: Peniophoraceae) with its phylogenetic analysis

**DOI:** 10.1080/23802359.2021.1945508

**Published:** 2021-07-05

**Authors:** Qiufen Gou, Chaoqin Ren, Cong Peng

**Affiliations:** a Leshan Vocational and Technical College, Leshan, Sichuan, China; b Aba Teachers University, Wenchuan, Sichuan, China; c School of Food and Biological Engineering, Chengdu University, Chengdu, Sichuan, China

**Keywords:** Mitochondrial genome, phylogenetic analysis, evolution

## Abstract

*Peniophora lycii* is a resupinate lichen-like species distributed all over the world. In the present study, we sequenced and assembled the complete mitochondrial genome of *Peniophora lycii*. The size of the mitochondrial genome of *P. lycii* was 38,296 bp, with a GC content of 25.89%. Twenty protein-coding genes, 2 ribosomal RNA genes, and 24 transfer RNA genes were identified in the mitochondrial genome of *P. lycii*. Phylogenetic analysis based on combined mitochondrial gene dataset indicated that the mitochondrial genome of *P. lycii* exhibited a close relationship with that of *Heterobasidion irregulare*.

The species *Peniophora lycii* (Pers.) Hohn. & Litsch. 1907 belongs to the Peniophoraceae family of the Russulales order. *P. lycii* is a resupinate lichen-like species that usually colonizes dead branches of deciduous and coniferous trees all over the world (Pontoppidan et al. 2007; Glazunova et al. 2020). The Russulales is a well-known order that contained morphologically diverse mushrooms (Miller et al. 2006). Species from this order have diverse lifestyles, including saprotrophic, ectomycorrhizal, root-parasitic, and insect-symbiotic (Geml et al. 2010; Zhou and Dai 2013). The family Peniophoraceae is primarily saprotrophic fungi. Mitochondrial genome has been widely used to understand the phylogeny, life pattern evolution and genetics of fungal species (Li et al. 2020a, 2020c; Wang et al. 2020a; Wu et al. 2021). However, up to now, no complete mitochondrial genome from the genus *Peniophora* has been reported. The mitochondrial genome of *P. lycii* will promote the understanding of the phylogeny, origin, and taxonomy of this important fungal genus.

The specimen (*P. lycii*) was collected from Sichuan, China (101.23 E; 27.56 N). A specimen was deposited in Collection Center of Chengdu University under the voucher number Ply_s93. The complete mitochondrial genome of *P. lycii* was sequenced and *de novo* assembled according to previous described methods (Li et al. 2019b; Cheng et al. 2021). Briefly, the total genomic DNA of *P. lycii* was extracted using a Fungal DNA Kit (D3390-00, Omega Bio-Tek, Norcross, GA). The extracted genomic DNA was purified using a Gel Extraction Kit (Omega Bio-Tek, Norcross, GA). We stored the purified DNA in Leshan Vocational and Technical College (No. DNA_ Ply_s93). Sequencing libraries were constructed for sequencing using a NEBNext® Ultra™ II DNA Library Prep Kit (NEB, Beijing, China). Whole genomic sequencing (WGS) of *P. lycii* was conducted using Illumina HiSeq 2500 Platform (Illumina, San Diego, CA). Illumina PCR adapter reads and low-quality reads from the paired-end were filtered using custom scripts. About 1.5% of low quality sequences were excluded from downstream analysis. The mitochondrial genome of *P. lycii* was assembled by NOVOPlasty v4.3.1 (Dierckxsens et al. 2017) using the *rns* gene of *Lactarius hatsudake* as the seed sequence (Li et al. 2019a). The average mitochondrial sequence coverage was 1357 ×. We annotated the complete mitochondrial genome of *P. lycii* according to previous described methods (Wang et al. 2020b; Ye et al. 2020). Briefly, the protein-coding genes, rRNA genes, tRNA genes, and introns of the *P. lycii* mitogenome were annotated using MITOS (Bernt et al. 2013) and MFannot (Valach et al. 2014), both based on the genetic code 4. The tRNA genes in the *P. lycii* mitochondrial genome were also predicted using tRNAscan-SE v1.3.1 (Lowe and Chan 2016). Different annotation results were verified and manual corrected according to the annotations of close related mitogenomes (Li et al. 2018).

The complete mitochondrial genome of *P. lycii* is 38,296 bp in length, which is the smallest mitochondrial genome in *Russulales* to date (Li et al. 2018, 2019a). The base composition of the *P. lycii* mitochondrial genome is as follows: A (36.80%), T (37.31%), G (12.87%) and C (13.01%). The complete mitochondrial genome of *P. lycii* contains 20 protein-coding genes, 2 ribosomal RNA genes (*rns* and *rnl*), and 24 transfer RNA genes. No intron was detected in the *P. lycii* mitochondrial genome (Zhang and Zhang 2019). We constructed a phylogenetic tree for 13 Russulales species to investigate the phylogenetic status of *P. lycii*. Bayesian analysis (BI) method was used to construct phylogenetic tree based on the combined 14 core protein-coding genes and 2 rRNA genes of mitochondrial genomes (*atp6*, *atp8*, *atp9*, *cob*, *cox1*, *cox2*, *cox3*, *nad1*, *nad2*, *nad3*, *nad4*, *nad4L*, *nad5*, *nad6*, *rns*, and *rnl*) according to previous described methods (Li et al. 2019c; 2020b; 2021b). First, we aligned individual protein-coding genes of mitochondrial genomes using MAFFT v7.037 (Katoh et al. 2019), and then we concatenated these alignments into a combined gene dataset using SequenceMatrix v1.7.8 (Vaidya et al. 2011). Potential phylogenetic conflicts between different genes were detected by a partition homogeneity test (Li et al. 2021a); PartitionFinder 2.1.1 (Lanfear et al. 2017) was used to determine best-fit models of evolution and partitioning schemes. MrBayes v3.2.0 (Ronquist et al. 2012) was used to perform the BI analysis. *Pleurotus cornucopiae* from the order Agaricales was set as outgroup (Xu et al. 2018). As shown in the phylogenetic tree (Figure 1), the mitochondrial genome of *P. lycii* exhibited a close relationship with that of *Heterobasidion irregular* (Himmelstrand et al. 2014).

**Figure 1.**
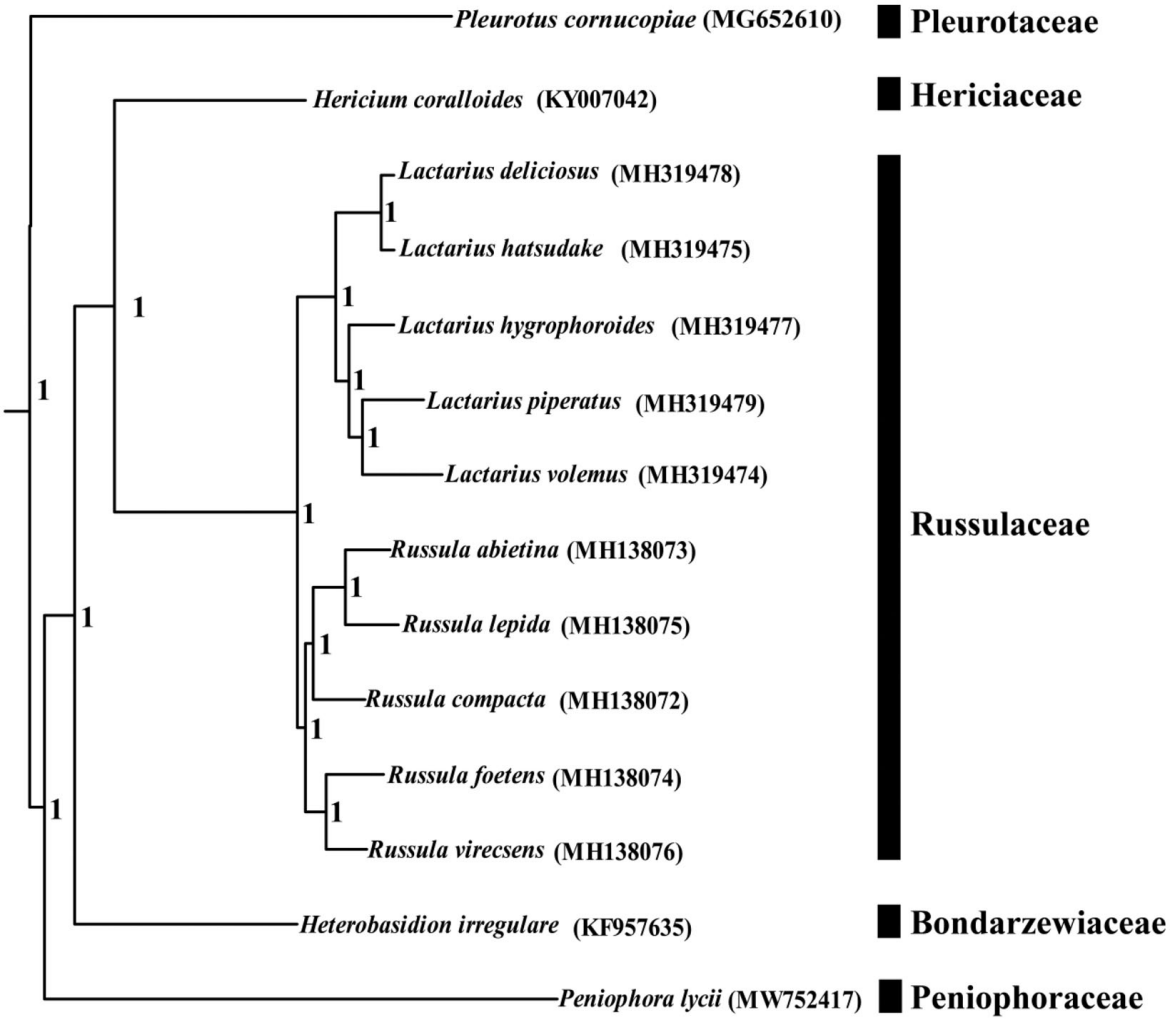
Bayesian phylogenetic analysis of 13 Russulales species. *Pleurotus cornucopiae* from the order Agaricales was set as outgroup. Accession numbers of mitochondrial genomes used in the phylogenetic analysis are listed in brackets.

## Data Availability

The genome sequence data that support the findings of this study are openly available in GenBank of NCBI at https://www.ncbi.nlm.nih.gov/ under the accession no. MW752417. The associated BioProject, SRA, and Bio-Sample numbers are PRJNA724896, SRR14320041, and SAMN18864199, respectively.

## References

[CIT0001] Bernt M, Donath A, Juhling F, Externbrink F, Florentz C, Fritzsch G, Putz J, Middendorf M, Stadler PF. 2013. MITOS: improved de novo metazoan mitochondrial genome annotation. Mol Phylogenet Evol. 69(2):313–319. 22982435 10.1016/j.ympev.2012.08.023

[CIT0002] Cheng J, Luo Q, Ren YH, Luo Z, Liao WL, Wang X, Li Q. 2021. Panorama of intron dynamics and gene rearrangements in the phylum Basidiomycota as revealed by the complete mitochondrial genome of *Turbinellus floccosus*. Appl Microbiol Biotechnol. 105(5):2017–2032. 33555361 10.1007/s00253-021-11153-w

[CIT0003] Dierckxsens N, Mardulyn P, Smits G. 2017. NOVOPlasty: de novo assembly of organelle genomes from whole genome data. Nucleic Acids Res. 45(4):e18. 28204566 10.1093/nar/gkw955 PMC5389512

[CIT0004] Geml J, Laursen GA, Herriott IC, McFarland JM, Booth MG, Lennon N, Nusbaum HC, Taylor DL. 2010. Phylogenetic and ecological analyses of soil and sporocarp DNA sequences reveal high diversity and strong habitat partitioning in the boreal ectomycorrhizal genus *Russula* (Russulales; Basidiomycota). New Phytol. 187(2):494–507. 20487310 10.1111/j.1469-8137.2010.03283.x

[CIT0005] Glazunova OA, Moiseenko KV, Savinova OS, Fedorova TV. 2020. Purification and characterization of two novel Laccases from *Peniophora lycii*. JoF. 6(4):340. 33291231 10.3390/jof6040340 PMC7762197

[CIT0006] Himmelstrand K, Olson A, Brandstrom Durling M, Karlsson M, Stenlid J. 2014. Intronic and plasmid-derived regions contribute to the large mitochondrial genome sizes of Agaricomycetes. Curr Genet. 60(4):303–313. 25011705 10.1007/s00294-014-0436-z PMC4201751

[CIT0007] Katoh K, Rozewicki J, Yamada KD. 2019. MAFFT online service: multiple sequence alignment, interactive sequence choice and visualization. Brief Bioinform. 20(4):1160–1166. 28968734 10.1093/bib/bbx108 PMC6781576

[CIT0008] Lanfear R, Frandsen PB, Wright AM, Senfeld T, Calcott B. 2017. PartitionFinder 2: new methods for selecting partitioned models of evolution for molecular and morphological phylogenetic analyses. Mol Biol Evol. 34(3):772–773. 28013191 10.1093/molbev/msw260

[CIT0009] Li Q, He X, Ren Y, Xiong C, Jin X, Peng L, Huang W. 2020a. Comparative mitogenome analysis reveals mitochondrial genome differentiation in ectomycorrhizal and asymbiotic Amanita species. Front Microbiol. 11:1382. 32636830 10.3389/fmicb.2020.01382 PMC7318869

[CIT0014] Li Q, Yang L, Xiang D, Wan Y, Wu Q, Huang W, Zhao G. 2020b. The complete mitochondrial genomes of two model ectomycorrhizal fungi (*Laccaria*): features, intron dynamics and phylogenetic implications. Int J Biol Macromol. 145:974–984. 31669472 10.1016/j.ijbiomac.2019.09.188

[CIT0030] Li Q, Ren Y, Xiang D, Shi X, Zhao J, Peng L, Zhao G. 2020c. Comparative mitogenome analysis of two ectomycorrhizal fungi (Paxillus) reveals gene rearrangement, introndynamics, and phylogeny of basidiomycetes. IMA Fungus. 11(1):12. 10.1186/s43008-020-00038-8 PMC7333402 32670777

[CIT0010] Li Q, Wang Q, Chen C, Jin X, Chen Z, Xiong C, Li P, Zhao J, Huang W. 2018. Characterization and comparative mitogenomic analysis of six newly sequenced mitochondrial genomes from ectomycorrhizal fungi (*Russula*) and phylogenetic analysis of the Agaricomycetes. Int J Biol Macromol. 119:792–802. 30076929 10.1016/j.ijbiomac.2018.07.197

[CIT0011] Li Q, Wang Q, Jin X, Chen Z, Xiong C, Li P, Liu Q, Huang W. 2019a. Characterization and comparative analysis of six complete mitochondrial genomes from ectomycorrhizal fungi of the *Lactarius* genus and phylogenetic analysis of the Agaricomycetes. Int J Biol Macromol. 121:249–260. 30308282 10.1016/j.ijbiomac.2018.10.029

[CIT0013] Li Q, Xiang D, Wan Y, Wu Q, Wu X, Ma C, Song Y, Zhao G, Huang W. 2019b. The complete mitochondrial genomes of five important medicinal *Ganoderma* species: features, evolution, and phylogeny. Int J Biol Macromol. 139:397–408. 31381907 10.1016/j.ijbiomac.2019.08.003

[CIT0029] Li Q, Ren Y, Shi X, Peng L, Zhao J, Song Y, Zhao G. 2019c. Comparative mitochondrial genome analysis of two ectomycorrhizal fungi (Rhizopogon) reveals dynamic changes of intron and phylogenetic relationships of the subphylum Agaricomycotina. IJMS. 20(20):5167. 10.3390/ijms20205167 PMC6829451 31635252

[CIT0012] Li Q, Wu P, Li L, Feng H, Tu W, Bao Z, Xiong C, Gui M, Huang W. 2021a. The first eleven mitochondrial genomes from the ectomycorrhizal fungal genus (*Boletus*) reveal intron loss and gene rearrangement. Int J Biol Macromol. 172:560–572. 33476615 10.1016/j.ijbiomac.2021.01.087

[CIT0028] Li Q, Li L, Feng H, Tu W, Bao Z, Xiong C, Wang X, Qing Y, Huang W. 2021b. Characterization of the complete mitochondrial genome of Basidiomycete yeast Hannaella oryzae: intron evolution, gene rearrangement and its phylogeny. Front Microbiol. 12:646567. 10.3389/fmicb.2021.646567 PMC8193148 34122362

[CIT0015] Lowe TM, Chan PP. 2016. tRNAscan-SE On-line: integrating search and context for analysis of transfer RNA genes. Nucleic Acids Res. 44(W1):W54–57. 27174935 10.1093/nar/gkw413 PMC4987944

[CIT0016] Miller SL, Larsson E, Larsson KH, Verbeken A, Nuytinck J. 2006. Perspectives in the new *Russulales*. Mycologia. 98(6):960–970. 17486972 10.3852/mycologia.98.6.960

[CIT0017] Pontoppidan K, Pettersson D, Sandberg AS. 2007. *Peniophora lycii* phytase is stable and degrades phytate and solubilises minerals in vitro during simulation of gastrointestinal digestion in the pig. J Sci Food Agric. 87(14):2700–2708. 20836179 10.1002/jsfa.3033

[CIT0018] Ronquist F, Teslenko M, van der Mark P, Ayres DL, Darling A, Hohna S, Larget B, Liu L, Suchard MA, Huelsenbeck JP. 2012. MrBayes 3.2: efficient Bayesian phylogenetic inference and model choice across a large model space. Syst Biol. 61(3):539–542. 22357727 10.1093/sysbio/sys029 PMC3329765

[CIT0019] Vaidya G, Lohman DL, Meier R. 2011. SequenceMatrix: concatenation software for the fast assembly of multi‐gene datasets with character set and codon information. Cladistics. 27(2):171–180. 34875773 10.1111/j.1096-0031.2010.00329.x

[CIT0020] Valach M, Burger G, Gray MW, Lang BF. 2014. Widespread occurrence of organelle genome-encoded 5S rRNAs including permuted molecules. Nucleic Acids Res. 42(22):13764–13777. 25429974 10.1093/nar/gku1266 PMC4267664

[CIT0021] Wang X, Song A, Wang F, Chen M, Li X, Li Q, Liu N. 2020a. The 206 kbp mitochondrial genome of *Phanerochaete carnosa* reveals dynamics of introns, accumulation of repeat sequences and plasmid-derived genes. Int J Biol Macromol. 162:209–219. 32562727 10.1016/j.ijbiomac.2020.06.142

[CIT0022] Wang X, Wang YJ, Yao W, Shen JW, Chen MY, Gao M, Ren JN, Li Q, Liu N. 2020b. The 256 kb mitochondrial genome of *Clavaria fumosa* is the largest among phylum Basidiomycota and is rich in introns and intronic ORFs. IMA Fungus. 11(1):26. 33292749 10.1186/s43008-020-00047-7 PMC7666478

[CIT0023] Wu P, Bao Z, Tu W, Li L, Xiong C, Jin X, Li P, Gui M, Huang W, Li Q. 2021. The mitogenomes of two saprophytic Boletales species (Coniophora) reveals intron dynamics and accumulation of plasmid-derived and non-conserved genes. Comput Struct Biotechnol J. 19:401–414. 33489009 10.1016/j.csbj.2020.12.041 PMC7804350

[CIT0024] Xu LM, Hinsinger DD, Jiang GF. 2018. The complete mitochondrial genome of the Basidiomycete fungus *Pleurotus cornucopiae* (Paulet) Rolland. Mitochondrial DNA Part B. 3(1):73–75. 33490488 10.1080/23802359.2017.1422405 PMC7800982

[CIT0025] Ye J, Cheng J, Ren Y, Liao W, Li Q. 2020. The first mitochondrial genome for Geastrales (*Sphaerobolus stellatus*) reveals intron dynamics and large-scale gene rearrangements of Basidiomycota. Front Microbiol. 11:1970. 32849488 10.3389/fmicb.2020.01970 PMC7432440

[CIT0026] Zhang S, Zhang YJ. 2019. Proposal of a new nomenclature for introns in protein-coding genes in fungal mitogenomes. IMA Fungus. 10(15):15. 32647619 10.1186/s43008-019-0015-5 PMC7325650

[CIT0027] Zhou LW, Dai YC. 2013. Taxonomy and phylogeny of wood-inhabiting hydnoid species in Russulales: two new genera, three new species and two new combinations. Mycologia. 105(3):636–649. 23360974 10.3852/12-011

